# Exercise motivation, physical exercise, and mental health among college students: examining the predictive power of five different types of exercise motivation

**DOI:** 10.3389/fpsyg.2024.1356999

**Published:** 2024-07-24

**Authors:** Jun Li, Lingjie Wang, Ling Pan, Ziao Hu, Ruiqiang Yin, Jia-Fu Liu

**Affiliations:** ^1^School of Design, Hainan Vocational University of Science and Technology, Haikou, China; ^2^Basic Course Department, Hengshui University, Hengshui, China; ^3^Physical Education College, Xianyang Normal University, Xianyang, China; ^4^School of Marxism, Guizhou Education University, Xianyang, China

**Keywords:** exercise motivation, physical exercise, mental health, college students, predictive power

## Abstract

**Introduction:**

The mental health (MH) of college students has long been a crucial concern for families, educational institutions, and society. Extensive research has demonstrated the influential role of exercise motivation in shaping MH. However, further investigation is warranted to ascertain which types of exercise motivation may have more influence on the MH of college students. The present study examined the direct effects of five distinct types of exercise motivation, namely health motivation (HM), appearance motivation (APM), fun motivation (FM), ability motivation (ABM), and social motivation (SM) on MH. Additionally, the study explored the potential mediating role of physical exercise (PE) in these relationships.

**Methods:**

An cross-sectional study design was employed. A total of 433 Chinese college students participated in the study and completed our questionnaires, which included the Exercise motivation scale (EM scale), the Physical exercise scale (PE scale), and the Mental health scale (MH scale).

**Results:**

The findings revealed a significant and positive relationship between all five categories of exercise motivation and the MH of college students. Specifically, FM was found to have the most pronounced impact on MH, followed by HM, ABM, SM, and APM, in descending order of influence. Furthermore, the impacts of HM, FM, ABM, and SM on MH were found to be partially mediated by PE. However, the association between APM and MH was entirely mediated by PE.

**Discussion:**

The present study contributes to enhancing the comprehension of the underlying mechanisms behind different exercise motivations in relation to PE and MH. Additionally, it offers practical implications for developing intervention strategies for improving the MH of college students.

## 1 Introduction

The period of university education is a critical transitional phase for adolescents before their integration into society. The prevalence of MH issues among college students has become increasingly common due to various factors such as alterations in living conditions and lifestyle, rigorous academic pursuits and internships, intricate interpersonal dynamics, and the challenging job market (Gao et al., 2020). According to a recent study conducted by Xu et al. (2024), the occurrence of mental illness among Chinese college students has reached a rate of 40.3%. Severe mental health issues among college students have a significant impact on their everyday lives, academic performance, professional endeavors, and social relationships. In more extreme circumstances, these problems can result in the development of mental disorders such as melancholy and anxiety and may even precipitate suicide (Ma et al., 2024). Hence, the expeditious and efficacious intervention for MH issues among college students emerges as a significant concern for educators.

According to Conrad (2020), privacy issues often deter college students from taking conventional psychological interventions offered by their universities, such as psychotherapy and counseling. PE has gained widespread attention and recognition in psychological interventions for college students as a rapid, effective, and easy-to-implement psychological intervention (Weinberg and Gould, 2023). Empirical studies have also shown that engaging in PE significantly and positively impacts college students’ mental well-being (Liu et al., 2024). Exercise motivation has been reported as a positive predictor of PE (Li et al., 2022) and MH (Allen and Jay, 2022). As classified as intrinsic motivation within the framework of self-determination theory, exercise motivation arises from an individual’s inherent desire to experience enjoyment and fulfillment through engaging in PE, which is a crucial factor influencing the mental well-being of young individuals (Granero-Jiménez et al., 2022). Empirical studies have shown that exercise motivation significantly affects an individual’s MH (Turner and Reed, 2022).

Previous research has provided valuable insights into the association between exercise motivation, PE, and MH benefits. However, these studies have predominantly examined exercise motivation as a broad construct, leaving unanswered questions regarding the specific types of exercise motivation that may have a more substantial influence on the MH of college students (Ntoumanis et al., 2021; Granero-Jiménez et al., 2022). While some research has examined the impact of individual dimensions of exercise motivation, these studies have primarily focused on their influence on PE (Litt et al., 2011; Li and Han, 2020). There remains to be a gap in the literature regarding the exploration of their influence on psychological well-being. Furthermore, a recent study conducted by Gomes et al. (2018) has revealed a significant disparity in behavioral performance predicted by exercise motivation among individuals with similar levels of exercise motivation. This discrepancy, commonly referred to as the motivation-behavior gap (Gomes et al., 2018), has been primarily attributed to other mediating or moderating factors in the aforementioned study, neglecting the potential influence of exercise motivation causes, including the disparity in predicting capacity across various forms of exercise motivation. Moreover, several studies have indicated that various forms of exercise motivation, including health motivation (HM), appearance motivation (APM), fun motivation (FM), ability motivation (ABM), and social motivation (SM), exert distinct impacts on individuals’ MH issues, such as anxiety and depressive symptoms (Zhang et al., 2019). Hence, it is imperative to conduct empirical studies to examine the impact of the five distinct forms of exercise motivation on college students’ PE and MH.

In brief, the primary purpose of this study is to investigate the predictive capacity of HM, APM, FM, ABM, and SM on MH and PE. Additionally, the study aims to compare the predictive power of these motivations to determine which type of exercise motivation holds greater predictive power. The secondary objective is to examine whether PE mediates the relationship between HM, APM, FM, ABM, SM, and MH.

## 2 Literature review

### 2.1 Exercise motivation

Exercise motivation refers to the underlying factors or perceived incentives that prompt individuals to initiate or sustain PE. Recent studies have demonstrated that exercise motivation plays a significant role in mitigating psychological stress (Kilpatrick et al., 2002; Granero-Jiménez et al., 2022). Chen et al. (2013) classified exercise motivation into five categories: HM, APM, FM, ABM, and SM. HM pertains to engaging in exercise to enhance physical well-being. APM involves exercising to improve body shape, manage weight, and enhance overall attractiveness. FM refers to the enjoyment and sense of accomplishment derived from exercise. ABM encompasses exercising to enhance personal skills and acquire new abilities. Lastly, SM involves exercising to foster friendships and establish new social connections. The five aforementioned categories collectively demonstrate that exercise motivation pertains to an individual’s inclination to fulfill their requirements related to health, aesthetics, enjoyment, competence, and social interaction through PE.

### 2.2 Five types of exercise motivation and MH

Previous studies have shown evidence for the predictive effect of exercise motivation as a comprehensive antecedent variable in relation to MH (Vella et al., 2021; Zhang et al., 2021). Exercise motivation falls under the category of intrinsic motivation, which refers to college students’ desire to derive emotional satisfaction from exercise. A positive psychological experience often accompanies exercise motivation. Furthermore, college students with solid exercise motivation are driven by the purpose of the exercise itself, as it can bring them emotional pleasure, which, in turn, positively impacts their mental health (Tang et al., 2020; Granero-Jiménez et al., 2022). The current study aims to investigate further the predictive power of the five distinct categories of exercise motivation in relation to the MH of college students. Liu et al. (2023) proposed that HM, APM, FM, ABM, and SM are all categorized as forms of intrinsic motivation. Intrinsic motivation for PE stems from an individual’s internal drive to experience enjoyment and fulfillment from exercise based on their inherent needs, which is crucial for enhancing the mental well-being of adolescents (Meyer et al., 2021). Furthermore, the study by Sundgot-Borgen et al. (2021) found a significant correlation between positive body image, high appearance confidence, and high levels of MH in college students. Based on the discussion above, we hypothesized that HM and APM could impact college students’ MH. Similarly, ABM and SM have been found to play a role in adolescents’ development of social skills, thereby fostering psychological well-being (Neuhaus et al., 2019). Hence, this study is about to investigate the potential positive influence of HM, APM, FM, ABM, and SM on the MH of college students, as well as to determine the extent of their predictive capabilities.

### 2.3 Five types of exercise motivation and PE

Previous studies have provided empirical evidence supporting the predictive significance of exercise motivation as a comprehensive factor in individual PE (Granero-Jiménez et al., 2022; Turner and Reed, 2022). Based on self-determination theory, exercise motivation falls under internal motivation. Individuals with high internal motivation will exert more effort and persistence in challenging activities and derive enjoyment and satisfaction, thus encouraging and strengthening their engagement in PE (Morris et al., 2022). Consequently, strong exercise motivation indicates college students’ active involvement and proactive participation in PE, which serves as a long-term motivator for their continued participation (Standage and Ryan, 2020). Prior studies have investigated the impact of five distinct forms of exercise motivation on PE. For instance, HM and APM were found to elicit a desire among college students to enhance their well-being and physical appearance through engaging in PE; FM was observed to heighten college students’ inclination towards the enjoyable sensations associated with PE; ABM and SM were identified as factors that fostered college students’ aspirations to acquire skills and establish social connections through the pursuit of PE; consequently, these various motivations collectively serve as driving forces that encourage college students to partake in PE (Li et al., 2022). The motivation for college students to participate in PE might stem from various purposes, including health promotion, image enhancement, enjoyment, skill development, and social interaction (Vicent et al., 2021). However, which type of exercise motivation may possess the most significant predictive capability for PE among college students has yet to be thoroughly investigated. Therefore, the current study is to assess the potential positive impact of HM, APM, FM, ABM, and SM on college students’ engagement in PE, as well as determine their respective predictive strengths.

### 2.4 PE and MH

According to Weinberg and Gould (2023), PE is a valuable MH intervention because it can yield prompt, efficient, affordable, and low-risk outcomes. Neurobiology studies have demonstrated that engaging in regular PE positively impacts the body’s release of dopamine and endorphins, thereby relieving tension and anxiety and effectively promoting MH (Sauers, 2023). Social psychology studies have discovered that PE reduce feelings of tension and loneliness among college students, fostering self-confidence and thereby positively impacting the mental well-being of these individuals (Biddle et al., 2019; Jakimanska, 2022). Empirical research has provided corroborating evidence regarding the psychological impacts of PE. For instance, Grasdalsmoen et al. (2020) conducted a study involving a sample of 5,054 college students, revealing a significant and negative relationship between PE and MH issues, including suicidal thoughts. This study further indicated that college students who did not engage in PE exhibited a nearly threefold rise in self-reported depression compared to their counterparts who regularly participated in PE. Rogowska et al. (2020) found in a sample of 1,512 college students that those who engaged in lower levels of PE exhibited significantly elevated levels of anxiety and depression compared to their counterparts who maintained regular PE routines. Consequently, the current study proposes that PE may benefit college students’ MH.

**Figure 1:**
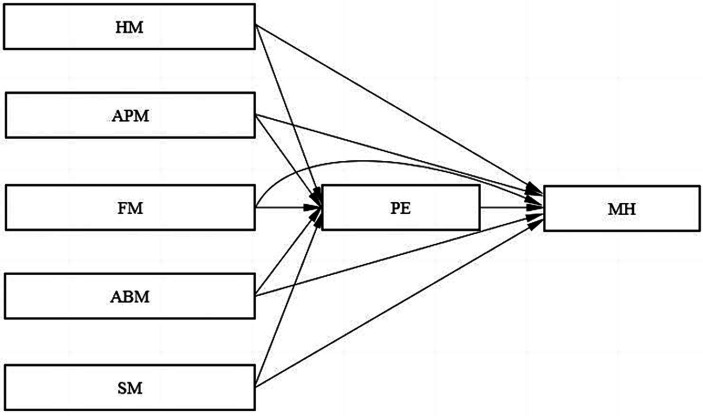
Conceptual model diagram. HM, health motivation; APM, appearance motivation; FM, fun motivation; ABM, ability motivation; SM, social motivation; PE, physical exercise; MH, mental health.

To summarize, HM, APM, FM, ABM, and SM may positively affect college students’ MH and PE. In addition, PE may also affect MH. Therefore, the following hypotheses are proposed (the conceptual model is shown in Figure 1):

*H1:* HM has a significant positive effect on MH;

*H2:* APM has a significant positive effect on MH;

*H3:* FM has a significant positive effect on MH;

*H4:* ABM has a significant positive effect on MH;

*H5:* SM has a significant positive effect on MH;

*H6:* PE has a mediating role between HM and MH;

*H7:* PE has a mediating role between APM and MH;

*H8:* PE has a mediating role between FM and MH;

*H9:* PE has a mediating role between ABM and MH;

*H10:* PE has a mediating role between SM and MH.

## 3 Methods

### 3.1 Participants and procedure

Given the time and human resource cost constraints, this study employed convenience sampling. Teachers who voluntarily participated in the distribution of questionnaires for this study were responsible for informing the participants of the contents of the consent form, which encompassing details regarding the voluntary aspect of participation, the assurance of anonymity and confidentiality of the questionnaire, and a reminder to participants that they possessed the right to decline or withdraw from the study at any given point. Following the participants’ provision of informed consent, the online questionnaire was completed and gathered using Questionnaire Star.^1^ Participants were given the option to voluntarily scan the QR code of the questionnaire if they expressed interest in participating in the study.

Based on the aforementioned methodology, a total of 553 questionnaires were gathered. After eliminating invalid questionnaires, 433 remained, resulting in a valid recovery rate of 78.3%. As per the sample size calculation method suggested by Ghiselli et al. (1981), the sample size should exceed 10 times the total number of questionnaire items. This study had 27 questionnaire items and 433 valid samples, satisfying the minimum sample size requirement. Among the valid samples, there were 187 (43.19%) male students, 246 (56.81%) female students, 115 (26.56%) non-only children, 318 (73.44%) only children, 118 (27.25%) first-year students, 123 (28.41%) second-year students, 101 (23.32%) third-year students, and 91 (21.02%) fourth-year students. The study had more female than male participants, which aligned with the demographic composition of Chinese college students (Huang et al., 2021). Additionally, 73.44% of the participants were only children, consistent with the distribution pattern observed in the Chinese “Post-00” generation, in which only children account for more than 60% (Li, 2019).

### 3.2 Research instruments

#### 3.2.1 Exercise motivation scale (EM scale)

The present study employed the EM scale, a psychometric tool developed by Chen et al. (2013). This scale was originally designed and validated using a sample of Chinese college students, demonstrating high reliability in previous studies. The scale comprises five distinct subscales that assess various dimensions of exercise motivation, including HM, APM, FM, ABM, and SM. The scale comprises 15 items, with each subscale encompassing 3 items (e.g., “I want to have a strong body”; refer to Table 1 for detailed question items). The responses were measured by a Likert 5-point scale ranging from 1 score (none) to 5 scores (very strong) for assessment of the level of motivation for each type of exercise. Each subscale’s total score was equal to the sum of each item’s score divided by the number of items (3 in the EM subscales case). Higher scores on each type of exercise were indicative of more motivation for that type.

**Table 1:** Items and validity analysis of each scale.

Dimension	Sequence number	Item	SFL	CR	AVE
HM	HM1	I want to have a strong body.	0.789	0.919	0.793
	HM2	I want to keep my body and mind healthy.	0.957		
	HM3	I want to live a healthy life.	0.916		
APM	APM1	I am trying to control my weight.	0.902	0.940	0.840
	APM2	I want to maintain or improve my body shape	0.964		
	APM3	I want to make my appearance more attractive.	0.882		
FM	FM1	I want to participate in entertaining activities	0.680	0.901	0.757
	FM2	I want to stay happy.	0.969		
	FM3	I want to have a good time.	0.933		
ABM	ABM1	I want to gain new athletic skills.	0.941	0.922	0.799
	ABM2	I want to improve my existing athletic skills	0.950		
	ABM3	I want to maintain my current level of athletic skill	0.781		
SM	SM1	I would like to meet some new people.	0.872	0.943	0.847
	SM2	I want to promote feelings and friendships with my friends	0.963		
	SM3	I want to maintain good social relations	0.924		
PE	PE1	It is hard for me to quit sports.	0.761	0.938	0.684
	PE2	I will have a strong desire to participate in physical activity after a few days of not exercising.	0.850		
	PE3	I have a hard time with the lack of physical exercise in my lifestyle	0.833		
	PE4	Physical exercise is an integral part of my life.	0.864		
	PE5	I am better at keeping up with my physical exercise.	0.851		
	PE6	I am in the habit of exercising.	0.843		
	PE7	I play a lot of sports.	0.782		
MH	MH1	I enjoy my life.	0.762	0.910	0.670
	MH2	I feel like my life has meaning.	0.783		
	MH3	I can focus on what I want to do, such as thinking, studying, memorizing, and so on.	0.830		
	MH4	I can live with my appearance.	0.870		
	MH5	I am happy with myself.	0.844		

HM, health motivation; APM, appearance motivation; FM, fun motivation; ABM, ability motivation; SM, social motivation; PE, physical exercise and MH, mental health; SFL, standardized factor loadings; CR, composite reliability; AVE, average variance extracted.

#### 3.2.2 Physical exercise scale (PE scale)

The current study employed the PE scale of Wu et al. (2016), which was initially established using a sample of Chinese college students and has demonstrated strong reliability. The measure comprises two dimensions, namely exercise commitment and exercise adherence. It encompasses 8 items, one of which is a reverse question. The matter pertaining to the impact of reverse questions on the dependability of the scale has garnered considerable attention. Extensive empirical and logical examinations have led researchers to deduce that the omission of reverse questions is a viable approach (Valdivia-Salas et al., 2021). Consequently, the reverse queries were excluded in the present study, and 7 elements remained (e.g., “It is hard for me to quit sports”; refer to Table 1 for detailed question items). The measurement instrument utilized a 5-point Likert scale to assess the level of engagement in PE, ranging from 1 score (representing entire disagreement) to 5 scores (representing complete agreement). In this study, PE was an overall variable with 2 dimensions, and the scale’s total score was equal to the sum of each item score divided by the number of items (which was 7 in the PE scale case). Higher scores on the scale were indicative of greater involvement in PE.

#### 3.2.3 Mental health scale (MH scale)

This study employed the World Health Organization quality of life assessment instrument brief version (WHOQOL-BREF), a MH scale that was adapted by the WHOQOL Group (1998). The scale has been extensively used in assessing the mental well-being of adolescents. Previous research has shown that the Chinese version of this scale has exhibited satisfactory reliability among Chinese college student populations (Lin, 2000). The scale comprises six items, one of which is a reversal question. In line with previous research on handling reverse questions (Valdivia-Salas et al., 2021), the current study opted to exclude reverse questions, leading to five remaining items (e.g., “I enjoy my life”; refer to Table 1 for detailed question items). The measurement instrument utilized a 5-point Likert scale, ranging from 1 score (completely disagree) to 5 scores (completely agree). The scale’s total score was equal to the sum of each item score divided by the number of items (5 in the MH scale case). Higher scores on the scale were indicative of greater levels of MH.

### 3.3 Statistical analysis

The data analysis in this study was conducted using SPSS 21.0. The significance of statistical significance was set at *p* < 0.05 throughout the data analysis process. Initially, descriptive statistics were performed on the participants’ background information, revealing the percentage distribution of participants. Next, the reliability tests were performed for each scale with Cronbach’s alpha exceeding 0.7, indicating that the measurement instrument was reliable (Nunnally, 1978). Thirdly, Harman’s one-factor test was conducted to determine common method variance (CMV). The unrotated factor analysis was performed, and if the explanatory power of the first factor did not surpass the crucial value of 50% (Podsakoff and Organ, 1986), the CMV problem was not significant. Fourth, descriptive statistics and correlation analysis were performed for each variable. Descriptive statistics reflected the means and standard deviations of each variable. Pearson’s correlations were employed to examine the relationships between variables with a correlation coefficient less than 0.8, indicating no colinearity issue and allowing for the execution of regression analysis (Benesty et al., 2009). Fifth, stratified regression analyses were performed to examine the direct impacts of each of the five exercise motivations on MH and the mediating role of PE in these direct associations.

Furthermore, AMOS 25.0 was utilized for the subsequent data analyses. First, to conduct confirmatory factor analysis (CFA). The criteria proposed by Hu and Bentler (1999) were used to determine if the measurement model fit was acceptable. The conditions for a satisfactory fit were as follows: the value of $\chi^{2}/df$ below 5, the root mean square residual (RMR) below 0.08, the standardized RMR (SRMR) below 0.08, the comparative fit index (CFI) above 0.80, the goodness-of-fit index (GFI) above 0.80, the parsimonious goodness-of-fit index (PGFI) above 0.50, the Tucker-Lewis index (TLI) above 0.80, and the incremental fit index (IFI) above 0.80. As per the criteria established by Bagozzi and Yi (1988), the presence of the following conditions implied a good convergent validity of scales: the standardized factor loading (SFL) above 0.5; the composite reliability (CR) above 0.6; the average variance extracted (AVE) above 0.4. Second, the fitness of the partial and complete mediation models was compared based on the abovementioned criteria established by Hu and Bentler (1999).

## 4 Results

### 4.1 Reliability and validity

The reliability analysis in this study yielded Cronbach’s alpha values were 0.910 for HM, 0.939 for APM, 0.879 for FM, 0.916 for ABM, 0.941 for SM, 0.937 for PE, and 0.910 for MH, demonstrating that the scales used in this study were of good reliability (Nunnally, 1978). This study utilized a multi-factor integrated model to assess the validity of each scale. Initially, a model fitness test was conducted through CFA. The results indicated that $\chi^{2}/df$ =4.060, SRMR = 0.056, RMR = 0.069, IFI = 0.919, TLI = 0.906, CFI = 0.919, GFI = 0.809, PGFI = 0.648, indicating a favorable fit of the model (Hu and Bentler, 1999). Furthermore, as depicted in Table 1, the SFL values for each question item on the scale varied between 0.680 and 0.969, CR values for each factor ranged from 0.901 to 0.943, and AVE values for each factor ranged from 0.670 to 0.847, indicating that a satisfactory convergent validity of the measurement model (Bagozzi and Yi, 1988).

### 4.2 Common method variance test

This study employed Harman’s one-factor test to assess common method variance (CMV). The test involved conducting an unrotated principal component factor analysis on the items of all variables. The analysis yielded a total of five factors, each with an eigenroot exceeding 1. Furthermore, the initial component accounted for 43.592% of the observed variation, which falls below the critical threshold of 50%. The findings suggest no substantial evidence of significant common technique bias issues in the present study (Podsakoff and Organ, 1986).

### 4.3 Descriptive statistics and correlations

This study conducted descriptive statistical analysis and correlation analysis to examine seven variables: HM, APM, FM, ABM, SM, PE, and MH. The findings are presented in Table 2, which displays the mean values of HM (*M* = 3.807), MH (*M* = 3.794), FM (*M* = 3.764), APM (*M* = 3.669), SM (*M* = 3.369), ABM (*M* = 3.356), PE (*M* = 3.172) in descending order, indicating that the participants in this study exhibited moderate to high levels of each variable. The correlation coefficients between the variables ranged from 0.136 to 0.724, suggesting a moderate-to-low level of correlation. These results also indicate no significant covariance issues among the variables (Benesty et al., 2009).

**Table 2:** Descriptive statistics and correlations.

Variable	*M*	*SD*	1	2	3	4	5	6	7
HM	3.807	0.964	1						
APM	3.669	1.152	0.517^***^	1					
FM	3.764	0.959	0.724^***^	0.573^***^	1				
ABM	3.356	1.060	0.629^***^	0.464^***^	0.696^***^	1			
SM	3.369	1.083	0.548^***^	0.460^***^	0.671^***^	0.671^***^	1		
PE	3.172	0.882	0.417^***^	0.251^***^	0.426^***^	0.586^***^	0.451^***^	1	
MH	3.794	0.880	0.359^***^	0.136^**^	0.402^***^	0.331^***^	0.323^***^	0.365^***^	1

^**^p < 0.01, ^***^p < 0.001.

HM, health motivation; APM, appearance motivation; FM, fun motivation; ABM, ability motivation; SM, social motivation; PE, physical exercise and MH, mental health; M, means; SD, standard deviations.

### 4.4 Regression analysis of five types of exercise motivation, PE and MH

**Figure 2:**
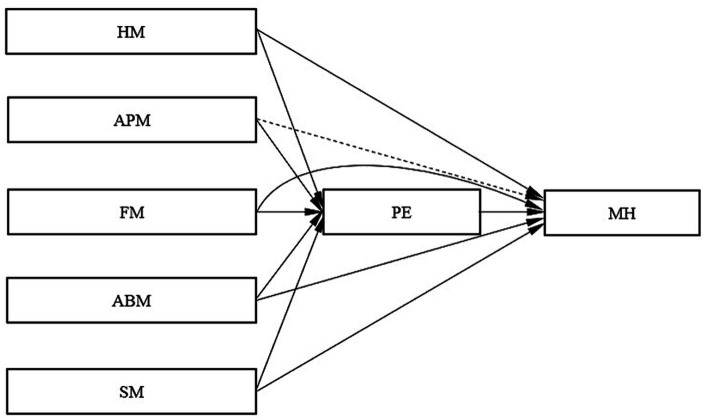
Path effect diagram. The solid line indicates that the path effect is significant, and the dotted line indicates that the path effect is not significant.

This study employed hierarchical regression analysis to investigate the predictive impact of HM, APM, FM, ABM, and SM on MH and PE. Additionally, the predictive influence of PE on MH was also examined (the effect diagram for each pathway is displayed in Figure 2). The data findings are presented in Table 3, wherein it is observed that each of the five categories of exercise motivation exhibited a statistically significant direct impact on MH in Model 1. Further analysis revealed that the predictive efficacy of the five categories of exercise motivation on MH was in descending order: FM (B = 0.402, *t* = 9.103***, R^2^ = 0.161) > HM (Β = 0.359, *t* = 7.977***, R^2^ = 0.129) >ABM (Β = 0.331, *t* = 7.294***, R^2^ = 0.110) > SM (Β = 0.323, *t* = 7.077***, R^2^ = 0.104) > APM (Β = 0.136, *t* = 2.858**, R^2^ = 0.019). These findings indicate that FM was the most robust predictor of MH among college students in the present study, while APM exhibits the weakest predictive power.

**Table 3:** Regression analysis of 5 motivation types, physical exercise and mental health.

	Model 1			Model 2			Model 3		
	MH			PE			MH		
	B (*t*)	R^2^	F	B (*t*)	R^2^	F	B (*t*)	R^2^	F
HM	0.359 (7.977^***^)	0.129	63.639^***^	0.417 (9.514^***^)	0.174	90.525^***^	0.250 (5.219^***^)	0.185	48.784^***^
PE							0.261 (5.449^***^)		
APM	0.136 (2.858^**^)	0.019	8.169^***^	0.251 (5.382^***^)	0.063	28.963^***^	0.048 (1.031)	0.135	33.682^***^
PE							0.353 (7.623^***^)		
FM	0.402 (9.103^***^)	0.161	82.859^***^	0.426 (9.768^***^)	0.181	95.420^***^	0.301 (6.335^***^)	0.207	56.220^***^
PE							0.237 (4.997^***^)		
ABM	0.331 (7.294^***^)	0.110	53.208^***^	0.586 (15.008^***^)	0.343	225.246^***^	0.179 (3.272^**^)	0.154	39.244^***^
PE							0.260 (4.755^***^)		
SM	0.323 (7.077^***^)	0.104	50.087^***^	0.451 (10.499^***^)	0.204	110.234^***^	0.198 (4.014^***^)	0.165	42.364^***^
PE							0.276 (5.580^***^)		

B are unstandardized coefficients; ^***^p < 0.001.

HM, health motivation; APM, appearance motivation; FM, fun motivation; ABM, ability motivation; SM, social motivation; PE, physical exercise; MH, mental health.

In model 2, it was found that all five categories of exercise motivation had a significant and positive impact on PE. A further examination of the predictive efficacy revealed that the five types of exercise motivation on PE were in descending order: ABM (Β = 0.586, t = 15.008***, R^2^ = 0.343) >SM (Β = 0.451, t = 10.499***, R^2^ = 0.204) >FM (Β = 0.426, t = 9.768***, R^2^ = 0.181) >HM (Β = 0.417, t = 9.514***, R^2^ = 0.174) >APM (Β = 0.251, t = 5.382**, R^2^ = 0.063). The findings suggested that ABM was the most robust predictor of PE among college students, while APM exhibits the weakest predictive power.

In model 3, PE was observed to significantly and positively impact MH after including PE as a mediating variable. However, the direct effect of APM on MH loses its significance. On the other hand, HM, FM, ABM, and SM continued to significantly and positively influence MH, albeit with a reduced magnitude compared to Model 1. The findings suggested that PE partially mediated the relationship between HM, FM, ABM, and SM on MH in the current study, while PE fully mediated the relationship between APM and MH.

Moreover, subsequent examination revealed that upon incorporating the mediating factor of PE, the explanatory rates of the five types of exercise motivation on PE were in descending order: FM (R^2^ = 0.207) > HM (R^2^ = 0.185) > SM (R^2^ = 0.165) >ABM (R^2^ = 0.154)>APM (R^2^ = 0.135). The findings implied that the factors of FM and PE demonstrate the highest explanatory power on MH among college students in the present study (R^2^ = 0.207).

### 4.5 Additional analysis: comparison of structural models

**Figure 3:**
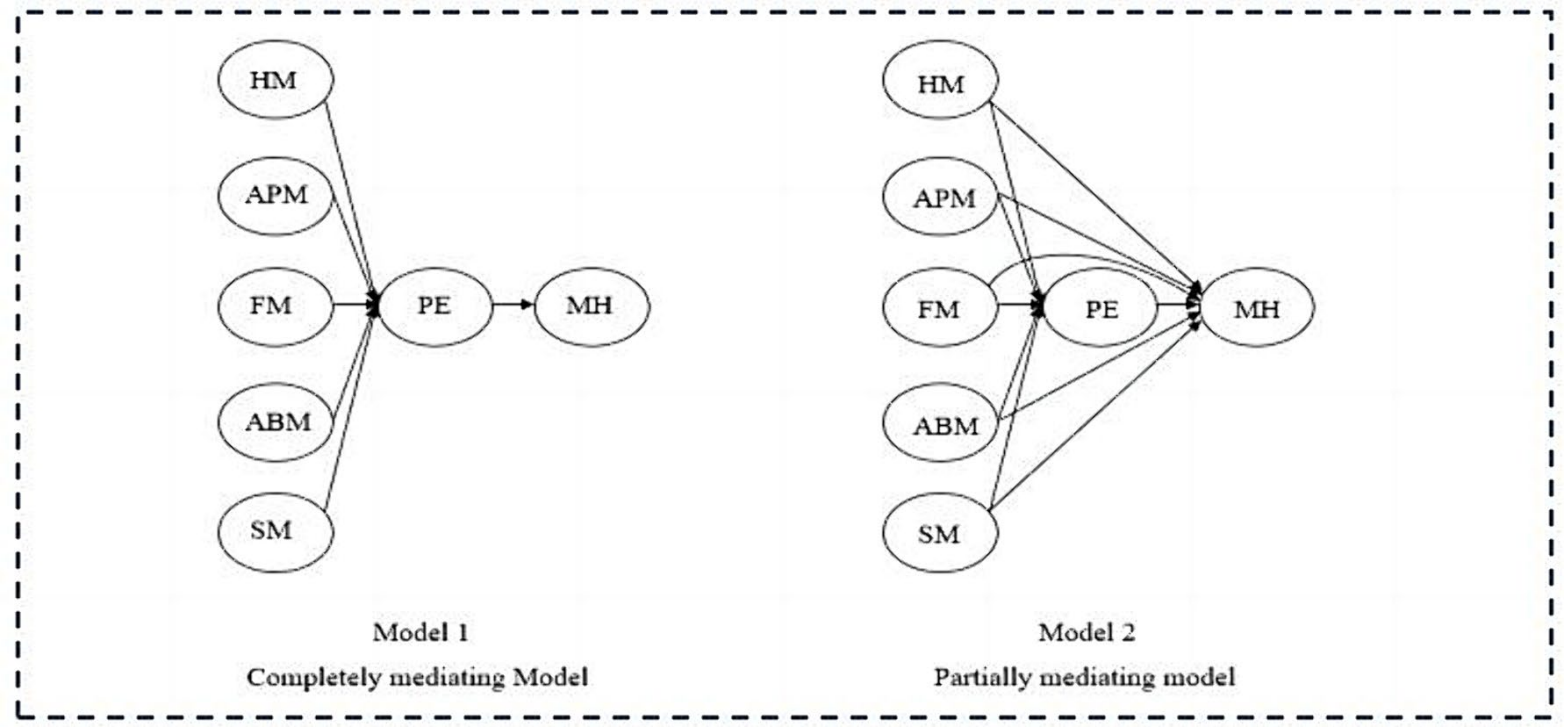
Model comparison chart. HM, health motivation; APM, appearance motivation; FM, fun motivation; ABM, ability motivation; SM, social motivation; PE, physical exercise; MH, mental health.

In order to evaluate which mediation model has the comparative performance between the partially mediated model and the completely mediated model, AMOS 25.0 software was employed to assess the fitness of the two types of mediation models, as depicted in Figure 3. According to Hu and Bentler (1998), the value of х^2^/ df should be less than 5, with smaller values indicating that the causal paths of the overall model are more compatible with the actual information. Hsiao et al. (2016) proposed that the RMSEA and RMR values should be less than 0.08, with smaller values indicating a more favorable model fit; the CFI, TLI, and IFI values should be greater than 0.9, with larger values indicating a better fit between the model and the data. Hence, as indicated in Table 4, both models provide a satisfactory fitness level. Subsequent comparisons indicate that the partially mediated model exhibited superior fit compared to the completely mediated model since its smaller *х^2^/ df*, RMSEA, and RMR values and its bigger CFI, TLI, and IFI values.

**Table 4:** Comparison of fit between fully mediated and partially mediated models.

Mediated model type	*χ^2^/ df*	RMR	CFI	TLI	IFI
Criteria	<5	<0.800	>0.900	>0.900	>0.900
Completely mediated model	4.143	0.094	0.915	0.903	0.915
Partial mediator model	4.060	0.069	0.919	0.906	0.919

*χ*^2^/ *df*, chi-square value/degree of freedom; RMR, root mean square residual; CFI, comparative fit index; TLI, Tucker–Lewis index; IFI, incremental fit index.

## 5 Conclusion and discussion

The regression analysis results indicate that HM, APM, FM, ABM, and SM have a significant and positive impact on MH. These findings support hypotheses H1, H2, H3, H4, and H5. First, HM refers to a psychological mindset encompassing self-awareness of one’s health and taking responsibility for it (Chen Y. et al., 2023). This mindset encourages individuals to introspect, gain self-understanding, and develop positive self-perceptions, contributing to a healthy psychological state (Alimour et al., 2023). Second, the positive impact of APM on MH can be explained by the theory of perfectionism dichotomy. According to this theory, individuals who have positive perfectionist traits are more likely to experience positive emotions and are less prone to negative emotional distress like anxiety and depression when pursuing expectations of success and competence. College students with APM tend to exhibit these positive perfectionist traits (Egan et al., 2007; Chernishenko et al., 2021). Third, individuals with FM are motivated by the enjoyment deriving from PE, as it fulfills their demand for pleasure. This experience can lead to happy emotions, enhance overall life satisfaction, and thus contribute to MH (Chen et al., 2013; Meyer et al., 2021). Fourth, individuals who possess a higher level of ABM are often accompanied by a greater sense of self-efficacy (Wang et al., 2015), and they tend to exhibit higher levels of psychological resilience, lower levels of anxiety and depression, and experience overall better health and happiness (Martínez-Martí and Ruch, 2017). This correlation helps to explain the positive impact of ABM on MH. Finally, a higher level of SM is indicative of healthy peer relationships. College students have the potential to cultivate new social abilities, get emotional support, and undergo favorable psychological encounters through interactions and exchanges with their peers (Smith, 2003; Scholte and Van Aken, 2020). According to self-determination theory, individuals generate HM, APM, FM, ABM, and SM based on their intrinsic needs. These motivations fall under the category of intrinsic motivation. Intrinsic motivation creates a state of mind where individuals seek pleasure and satisfaction from exercise, leading to positive psychological experiences (Granero-Jiménez et al., 2022; Liu et al., 2023).

This study also discovered that PE served as a partial mediator between HM, FM, ABM, SM, and MH. Additionally, PE acted as a complete mediator between APM and MH, confirming hypotheses H6, H7, H8, H9, and H10. According to the definition of the five types of exercise motivation, college students with high levels of HM, APM, FM, ABM, and SM have a strong desire to fulfill their needs related to promoting health, improving body shape, satisfying interests, enhancing abilities, and expanding interpersonal relationships through specific behaviors (Chen et al., 2013). These needs align with the fundamental purposes of engaging in PE, which include improving abilities, enhancing physical health, and enriching cultural experiences for students (Chen S. P. et al., 2023). Therefore, the positive effects of the five types of exercise motivation on PE can be explained. Furthermore, the positive impact of PE on MH can be attributed to physiological and psychological factors. PE stimulates the body to produce higher levels of dopamine and endorphins, reducing feelings of tension and anxiety. Additionally, PE aids in the development of stronger social connections and boosts self-confidence, ultimately leading to improved MH among college students (Biddle et al., 2019; Sauers, 2023). Besides, it is crucial to emphasize that the impact of APM on MH can be attributed to the pursuit of weight control and body improvement through PE among college students, as highlighted by Chen et al. (2013). This endeavor not only fosters self-confidence but also contributes to MH promotion (Sundgot-Borgen et al., 2021). College students who solely possess APM without actively engaging in PE are unable to attain the objective of weight management and body enhancement, thereby failing to foster MH. Therefore, PE acts as a complete mediator between APM and MH.

This study compared the predictive power of the five exercise motivations and determined that FM and HM were more predictive of MH, both directly and through the mediation of PE. Typically, an individual with robust exercise motivation exhibits a pronounced inclination towards engaging in PE, driven by a combination of enjoyment, the pursuit of well-being, and a propensity to engage in regular exercise to fulfill various personal needs. This motivation serves to enhance both their physical self-perception and overall self-worth, thereby fostering psychological well-being (Dong and Mao, 2020). The inclination of college students to seek enjoyment in PE to alleviate pressure, regulate emotions, and promote psychological well-being may be attributed to the multifaceted pressures they face in academics, interpersonal communication, employment, and other domains (Ji and Zheng, 2021; Zhu et al., 2021). Furthermore, there is a widely accepted sense that engaging in PE can enhance physical health, which in turn can bolster college students’ self-confidence and self-esteem, both of which are intricately linked to their MH (Biddle et al., 2019).

Through a comparison of the predictive power of the five exercise motivations, it was determined that ABM exhibited the strongest predictive strength, whereas APM demonstrated the weakest. This phenomenon may be attributed to universities’ emphasis on developing and assessing students’ overall qualities. Besides acquiring knowledge in various academic disciplines, college students are also expected to showcase and validate their abilities through physical activities (Chu et al., 2009). This emphasis on PE enhances the predictive influence of motivation based on ability. Furthermore, in recent years, the beauty and cosmetic industry, including medical cosmetology, plastic surgery, and artificial weight-loss establishments, has targeted college students as a significant consumer group. These institutions have extensively advertised their services within colleges and universities to attract customers (Alotaibi, 2021). Consequently, more college students may opt for these technological interventions to enhance their appearance rather than engage in PE. As a result, the impact of appearance motivation on college students’ participation in PE is weakened.

## 6 Practical implications

The current study examined the direct impacts of the five distinct forms of exercise motivation on MH, as well as their indirect influences on MH through the mediation of PE. The results have ultimately identified that among the five distinct forms of exercise motivation on MH, FM and HM serve as the primary factors, directly and indirectly, influencing MH. The current study’s findings suggest that college educators prioritize the enhancement of college students’ FM and HM to effectively utilize exercise motivation and PE as interventions for improving college students’ MH. One potential strategy to increase college students’ FM is implementing competitive sports events and enjoyable activities. For example, diversified physical activities can be developed based on college students’ interests, including parkour, biking, swimming, and rock climbing (Wintle, 2022). Furthermore, in the contemporary era of mobile Internet, it is possible to guide college students regarding using electronic sports equipment and software, such as sports watches and sports applications, to augment the pleasure derived from engaging in PE (Bitrián et al., 2020). Additionally, incorporating theoretical courses into physical education can augment college students’ understanding of the significance of PE in promoting physical well-being, and enhance their HM. By bolstering college students’ FM and HM, ultimately advancing their MH.

## 7 Limitations and directions for future research

Several limitations are manifested in this investigation. Initially, the data utilized in this study was obtained solely through self-reports provided by college students. While Harman’s one-way test indicated no severe problem with CMV, it is recommended for future research endeavors to incorporate a diverse range of methodologies, including interviews and observations. This approach will mitigate the inherent bias of relying solely on a single research method. Furthermore, the present study employed cross-sectional data, which depicts the association of each variable at a specific moment and does not prove a causal relationship between such variables. While some scholars have contended that cross-sectional studies offer valuable insights into the associations between variables (Kline, 2015; Hayes, 2017), it is suggested that subsequent investigations corroborate the findings of this study using longitudinal data.

## Data availability statement

The data analyzed in this study is subject to the following licenses/restrictions: the raw data supporting the conclusions of this article will be made available by the authors, without undue reservation. Requests to access these datasets should be directed to LP, panling.edu.ma@foxmail.com.

## Ethics statement

The studies involving humans were approved by the ethics review board of Hainan Vocational University of Science and Technology (HKD-2022-30). The studies were conducted in accordance with the local legislation and institutional requirements. The participants provided their written informed consent to participate in this study.

## Author contributions

JL: Writing – review & editing, Writing – original draft, Methodology, Conceptualization. LW: Writing – review & editing, Writing – original draft, Validation, Software, Conceptualization. LP: Writing – review & editing, Validation, Investigation, Data curation. ZH: Writing – review & editing, Validation, Investigation, Data curation. RY: Writing – review & editing, Investigation, Data curation. J-FL: Writing – review & editing, Validation.

## Footnotes

^1^ www.wjx.cn

## References

[ref1] Alimour S. A. Rish A. Abu M. A. Alqawasmi A. A. (2023). Exploring role of metacognitive components and their awareness in mental health behavior among students of higher educational institutions. Am. J. Health Behav. 47, 904–917. doi: 10.5993/AJHB.47.5.4

[ref2] Allen D. Jay B. (2022). OPAL: Promoting mental health through friendship, socialisation and physical activity. Project Report, Manchester Metropolitan University. Available at: https://e-space.mmu.ac.uk/id/eprint/630413

[ref3] Alotaibi A. S. (2021). Demographic and cultural differences in the acceptance and pursuit of cosmetic surgery: a systematic literature review. Plast. Reconstr. Surg. Glob. Open 9, e3501–e3507. doi: 10.1097/GOX.0000000000003501, PMID: 33777604 PMC7990019

[ref4] Bagozzi R. P. Yi Y. (1988). On the evaluation of structural equation models. J. Acad. Market Sci. 16, 74–94. doi: 10.1007/BF02723327

[ref5] Benesty J. Chen J. Huang Y. Cohen I. (2009). Noise reduction in speech processing. New York: Springer.

[ref6] Biddle S. J. H. Ciaccioni S. Thomas G. Vergeer I. (2019). Physical activity and mental health in children and adolescents: an updated review of reviews and an analysis of causality. Psychol. Sport Exerc. 42, 146–155. doi: 10.1016/j.psychsport.2018.08.011

[ref7] Bitrián P. Buil I. Catalán S. (2020). Gamification in sport apps: the determinants of users' motivation. Eur. J. Manag. Bus. Econ. 29, 365–381. doi: 10.1108/EJMBE-09-2019-0163

[ref9] Chen S. P. Song D. Xie L. J. Zhang Z. J. Liu L. P. (2023). Clustering analysis of college students’ exercise motivation and physical behavior characteristics. J. Cap. Univ. Phys. Educ. Sports 35, 57–67. doi: 10.14036/j.cnki.cn11-4513.2023.01.007

[ref10] Chen S. P. Wang Y. B. Rong J. Z. Pan X. G. Bao J. (2013). The simplified version of the MPAM-R: reliability and validity. J. Beijing Sport Univ. 36, 66–70. doi: 10.19582/j.cnki.11-3785/g8.2013.02.013

[ref8] Chen Y. Li S. Chen Y. W. (2023). Impact of overwork on turnover intention: based on the perspective of health risk perception. Chin. J. Clin. Psychol. 31, 208–212. doi: 10.16128/j.cnki.1005-3611.2023.01.039

[ref11] Chernishenko J. Mesler R. M. Basil D. Z. (2021). I can be perfect! Implicit mindset moderates the relationship between perfectionism and consumers' maladaptive weight management behavior. Personal. Individ. Differ. 183:111084. doi: 10.1016/j.paid.2021.111084

[ref12] Chu Y. D. Jin W. H. Wang Y. C. (2009). Relationship between college students’ exercise motivation and the motivation and sticking exercise. J. Beijing Sport Univ. 32, 85–97. doi: 10.19582/j.cnki.11-3785/g8.2009.03.024

[ref9001] Conrad R. (2020). Legal and ethical challenges in the psychiatric treatment of college students. Current psychiatry reports, 22, 1–4. doi: 10.1007/s11920-020-01168-x 32666191

[ref13] Dong B. L. Mao L. J. (2020). Exercise identity, internal motivation, exercise commitment and undergraduates' exercise behavior: a model of chain mediating effect. J. Tianjin Univ. Sport 35, 415–422. doi: 10.13297/j.cnki.issn1005-0000.2020.04.008

[ref14] Egan S. J. Piek J. P. Dyck M. J. Rees C. S. (2007). The role of dichotomous thinking and rigidity in perfectionism. Behav. Res. Ther. 45, 1813–1822. doi: 10.1016/j.brat.2007.02.002, PMID: 17382290

[ref15] Gao L. Xie Y. Jia C. Wang W. (2020). Prevalence of depression among Chinese university students: a systematic review and meta-analysis. Sci. Rep. 10:15897. doi: 10.1038/s41598-020-72998-1, PMID: 32985593 PMC7522998

[ref9003] Ghiselli EE Campbell JP Zedeck S (1981). Measurement theory for the behavioral sciences. W. H. Freeman, San Francisco. Available at: http://pascal-francis.inist.fr/vibad/index.php?action=getRecordDetail\u0026amp;idt=12541408.

[ref16] Gomes A. R. Gonçalves A. M. Maddux J. E. Carneiro L. (2018). The intention-behaviour gap: an empirical examination of an integrative perspective to explain exercise behaviour. Int. J. Sport Exerc. Psychol. 16, 607–621. doi: 10.1080/1612197X.2017.1321030

[ref17] Granero-Jiménez J. López-Rodríguez M. M. Dobarrio-Sanz I. Cortés-Rodríguez A. E. (2022). Influence of physical exercise on psychological well-being of young adults: a quantitative study. Int. J. Environ. Res. Public Health 19:4282. doi: 10.3390/ijerph19074282, PMID: 35409963 PMC8998469

[ref18] Grasdalsmoen M. Eriksen H. R. Lønning K. J. Sivertsen B. (2020). Physical exercise, mental health problems, and suicide attempts in university students. BMC Psychiatry 20, 175–111. doi: 10.1186/s12888-020-02583-3, PMID: 32299418 PMC7164166

[ref19] Hayes A. F. (2017). Introduction to mediation, moderation, and conditional process analysis: A regression-based approach. New York: Guilford Publications.

[ref21] Hsiao C. Lee Y. H. Chen H. H. (2016). The effects of internal locus of control on entrepreneurship: the mediating mechanisms of social capital and human capital. Int. J. Hum. Resour. Manage. 27, 1158–1172. doi: 10.1080/09585192.2015.1060511

[ref24] Huang Y. Su X. Si M. Xiao W. Wang H. Wang W.. (2021). The impacts of coping style and perceived social support on the mental health of undergraduate students during the early phases of the COVID-19 pandemic in China: a multicenter survey. BMC Psychiatry 21:530. doi: 10.1186/s12888-021-03546-y, PMID: 34706690 PMC8549419

[ref22] Hu L. T. Bentler P. M. (1998). Fit indices in covariance structure modeling: sensitivity to underparameterized model misspecification. Psychol. Methods 3, 424–453. doi: 10.1037//1082-989X.3.4.424

[ref23] Hu L. T. Bentler P. M. (1999). Cutoff criteria for fit indexes in covariance structure analysis: conventional criteria versus new alternatives. Struct. Equ. Model. 6, 1–55. doi: 10.1080/10705519909540118

[ref25] Jakimanska I. S. (2022). Psychological features of self-attitude of teenagers engaged in sports. Psychologist 2, 39–50. doi: 10.25136/2409-8701.2022.2.37571

[ref26] Ji H. Zheng C. (2021). The influence of physical exercise on college students’ mental health and social adaptability from the cognitive perspective. Work 69, 651–662. doi: 10.3233/WOR-213506, PMID: 34120942

[ref28] Kilpatrick M. Hebert E. Jacobsen D. (2002). Physical activity motivation: a practitioner's guide to self-determination theory. J. Phys. Educ. Recreat. Dance 73, 36–41. doi: 10.1080/07303084.2002.10607789

[ref29] Kline R. B. (2015). The mediation myth. Basic Appl. Soc. Psychol. 37, 202–213. doi: 10.1080/01973533.2015.1049349

[ref32] Li B. Han S. Meng S. Lee J. Cheng J. Liu Y. (2022). Promoting exercise behavior and cardiorespiratory fitness among college students based on the motivation theory. BMC Public Health 22:738. doi: 10.1186/s12889-022-13159-z, PMID: 35418043 PMC9009046

[ref30] Li C. L. (2019). Children born in the era of reform and opening-up: China's new generation and a new era of development. Sociol. Stud. 34, 1–24. doi: 10.19934/j.cnki.shxyj.2019.03.001

[ref33] Lin M. R. (2000). Introduction to the development of the WHOQOL-Taiwan version. Chin. J. Public Health 19, 315–324. doi: 10.6288/CJPH2000-19-04-10

[ref34] Litt D. M. Iannotti R. J. Wang J. (2011). Motivations for adolescent physical activity. J. Phys. Activity Health 8, 220–226. doi: 10.1123/jpah.8.2.220 21415449

[ref35] Liu L. Chen S. Yang X. Yang Y. (2023). Family and social class differences in sports behavior motivation among college students: an empirical study based on the latent class model. Front. Psychol. 14:1070862. doi: 10.3389/fpsyg.2023.1070862, PMID: 36760445 PMC9905112

[ref36] Liu R. Menhas R. Saqib Z. A. (2024). Does physical activity influence health behavior, mental health, and psychological resilience under the moderating role of quality of life? Front. Psychol. 15:1349880. doi: 10.3389/fpsyg.2024.1349880, PMID: 38529092 PMC10961448

[ref31] Li Y. X. Han S. S. (2020). A study of college students' exercise behavior and aerobic fitness promotion pathways. Chin. J. Sch. Health 41, 1270–1272. doi: 10.16835/j.cnki.1000-9817.2020.08.042

[ref38] Martínez-Martí M. L. Ruch W. (2017). Character strengths predict resilience over and above positive affect, self-efficacy, optimism, social support, self-esteem, and life satisfaction. J. Posit. Psychol. 12, 110–119. doi: 10.1080/17439760.2016.1163403

[ref37] Ma S. Yang Y. Soh K. G. Tan H. (2024). Effects of physical fitness on mental health of Chinese college students: across-sectional study. BMC Public Health 24, 727–729. doi: 10.1186/s12889-024-18097-6, PMID: 38448880 PMC10918864

[ref39] Meyer S. Grob A. Gerber M. (2021). No fun, no gain: the stress-buffering effect of physical activity on life satisfaction depends on adolescents' intrinsic motivation. Psychol. Sport Exerc. 56:102004. doi: 10.1016/j.psychsport.2021.102004

[ref9002] Morris L. S. Grehl M. M. Rutter S. B. Mehta M. Westwater M. L. (2022). On what motivates us: A detailed review of intrinsic v. extrinsic motivation. Psychological medicine, 52, 1801–1816. doi: 10.1017/S0033291722001611 35796023 PMC9340849

[ref40] Neuhaus E. Webb S. J. Bernier R. A. (2019). Linking social motivation with social skill: the role of emotion dysregulation in autism spectrum disorder. Dev. Psychopathol. 31, 931–943. doi: 10.1017/S0954579419000361, PMID: 30957732 PMC8148424

[ref41] Ntoumanis N. Ng J. Y. Prestwich A. Quested E. Hancox J. E. Thøgersen-Ntoumani C.. (2021). A meta-analysis of self-determination theory-informed intervention studies in the health domain: effects on motivation, health behavior, physical, and psychological health. Health Psychol. Rev. 15, 214–244. doi: 10.1080/17437199.2020.1718529, PMID: 31983293

[ref42] Nunnally J. C. (1978). “An overview of psychological measurement” in Clinical diagnosis of mental disorders. ed. Wolman B. B. (Boston, MA: Springer), 97–146.

[ref43] Podsakoff P. M. Organ D. W. (1986). Self-reports in organizational research: problems and prospects. J. Manage. 12, 531–544. doi: 10.1177/014920638601200408

[ref44] Rogowska A. M. Pavlova I. Kuśnierz C. Ochnik D. Bodnar I. Petrytsa P. (2020). Does physical activity matter for the mental health of university students during the COVID-19 pandemic? J. Clin. Med. 9:3494. doi: 10.3390/jcm9113494, PMID: 33138047 PMC7693909

[ref45] Sauers E. (2023). *Improving mental health through exercise.* Kines – 4350: Senior seminar in kinesiology, Our Lady of the Lake University. Available at: https://www.researchgate.net/publication/368719669

[ref46] Scholte R. H. Van Aken M. A. (2020). “Peer relations in adolescence” in Handbook of adolescent development. eds. Jackson S. Goossens L. (London: Psychology Press), 175–199.

[ref47] Smith A. L. (2003). Peer relationships in physical activity contexts: a road less traveled in youth sport and exercise psychology research. Psychol. Sport Exerc. 4, 25–39. doi: 10.1016/S1469-0292(02)00015-8

[ref48] Standage M. Ryan R. M. (2020). “Chapter 3 self-determination theory in sport and exercise” in Handbook of sport psychology. eds. Tenenbaum G. Eklund R. C. (New York: Wiley Online Library), 37–56.

[ref49] Sundgot-Borgen C. Sundgot-Borgen J. Bratland-Sanda S. Kolle E. Torstveit M. K. Svantorp-Tveiten K. M.. (2021). Body appreciation and body appearance pressure in Norwegian university students comparing exercise science students and other students. BMC Public Health 21, 532–511. doi: 10.1186/s12889-021-10550-0, PMID: 33740918 PMC7977603

[ref50] Tang M. Wang D. Guerrien A. (2020). A systematic review and meta-analysis on basic psychological need satisfaction, motivation, and well-being in later life: contributions of self-determination theory. PsyCh J. 9, 5–33. doi: 10.1002/pchj.293, PMID: 31177644

[ref51] Turner A. R. Reed S. M. (2022). Intrinsic motivation in exercise: a concept analysis. Nursing Forum 57, 136–143. doi: 10.1111/nuf.12658 34558057

[ref52] Valdivia-Salas S. Jiménez T. I. Lombas A. S. López-Crespo G. (2021). School violence towards peers and teen dating violence: the mediating role of personal distress. Int. J. Environ. Res. Public Health 18:310. doi: 10.3390/ijerph18010310, PMID: 33406621 PMC7795813

[ref53] Vella S. A. Benson A. Sutcliffe J. McLaren C. Swann C. Schweickle M. J.. (2021). Self-determined motivation, social identification and the mental health of adolescent male team sport participants. J. Appl. Sport Psychol. 33, 452–466. doi: 10.1080/10413200.2019.1705432

[ref54] Vicent M. Sanmartín R. Gonzálvez C. Vásconez-Rubio O. García-Fernández J. M. (2021). Perfectionism, motives, and barriers to exercise from a person-oriented approach. Int. J. Environ. Res. Public Health 18:8125. doi: 10.3390/ijerph18158125, PMID: 34360418 PMC8345606

[ref55] Wang Z. Hu G. P. Cai Y. J. Zhang L. (2015). Effect of procrastination on physical exercise motivation of college students: a mediating effect of self-efficacy. J. B. Sport Univ. 38, 71–77. doi: 10.19582/j.cnki.11-3785/g8.2015.04.012

[ref56] Weinberg R. S. Gould D. (2023). Foundations of sport and exercise psychology. Champaign, IL: Human Kinetics.

[ref57] WHOQOL Group (1998). Development of the World Health Organization WHOQOL-BREF quality of life assessment. Psychol. Med. 28, 551–558. doi: 10.1017/S0033291798006667 9626712

[ref58] Wintle J. (2022). Physical education and physical activity promotion: lifestyle sports as meaningful experiences. Educ. Sci. 12:181. doi: 10.3390/educsci12030181

[ref59] Wu Z. Y. Mao Z. X. Guo L. (2016). Development of psychological decision-making model of exercise adherence: the value added contribution of positive affective experience. J. Tianjin Univ. Sport 31, 77–81. doi: 10.13297/j.cnki.issn1005-0000.2016.01.015

[ref60] Xu B. Chen C. Wang D. (2024). Current psychotic-like experiences among Chinese college students: prevalence, correlates, and its relationship with suicidal ideation. Psychol. Res. Behav. Manag. 17, 799–811. doi: 10.2147/PRBM.S451889, PMID: 38434958 PMC10908336

[ref61] Zhang J. Gu X. Zhang X. Lee J. Chang M. Zhang T. (2021). Longitudinal effects of motivation and physical activity on depressive symptoms among college students. Int. J. Environ. Res. Public Health 18:5121. doi: 10.3390/ijerph18105121, PMID: 34065999 PMC8151539

[ref62] Zhang R. Pan T. Yang L. Liu P. (2019). The effect of different exercise motivation levels on short-term emotional benefit of physical exercise. Chin. J. Sports Med. 38, 864–873. doi: 10.16038/j.1000-6710.2019.10.007

[ref63] Zhu X. Haegele J. A. Liu H. Yu F. (2021). Academic stress, physical activity, sleep, and mental health among Chinese adolescents. Int. J. Environ. Res. Public Health 18:7257. doi: 10.3390/ijerph18147257, PMID: 34299708 PMC8304898

